# Cryptococcal Antigenemia in Human Immunodeficiency Virus Antiretroviral Therapy–Experienced Ugandans With Virologic Failure

**DOI:** 10.1093/cid/ciz1069

**Published:** 2019-11-03

**Authors:** Edward Mpoza, Radha Rajasingham, Lillian Tugume, Joshua Rhein, Maria Sarah Nabaggala, Isaac Ssewanyana, Wilson Nyegenye, Grace Esther Kushemererwa, Vivienne Mulema, Julius Kalamya, Charles Kiyaga, Joseph Kabanda, Mina Ssali, David R Boulware, David B Meya

**Affiliations:** 1 Infectious Diseases Institute, Makerere University, Kampala, Uganda; 2 Division of Infectious Diseases and International Medicine, Department of Medicine, University of Minnesota, Minneapolis, Minnesota, USA; 3 Uganda National Health Laboratory Systems, Kampala, Uganda; 4 Clinton Health Access Initiative, Uganda; 5 Centers for Diseases Control and Prevention - Uganda, Entebbe, Uganda; 6 Ministry of Health, Kampala, Uganda; 7 School of Medicine, College of Health Sciences, Makerere University, Kampala, Uganda

**Keywords:** cryptococcal antigenemia, virologic failure, ART experienced, HIV

## Abstract

**Background:**

Detectable serum or plasma cryptococcal antigen (CrAg) precedes symptomatic cryptococcal meningitis. The World Health Organization recommends CrAg screening for human immunodeficiency virus–positive persons with CD4 count <100 cells/μL initiating antiretroviral therapy (ART). However, an increasing proportion of patients with cryptococcosis are now ART experienced. Whether CrAg screening is cost-effective in those with virologic failure is unknown.

**Methods:**

We retrospectively performed nationwide plasma CrAg testing among ART-experienced Ugandan adults with virologic failure (≥1000 copies/mL) using leftover plasma after viral load testing during September 2017–January 2018. For those who were CrAg positive, we obtained ART history, meningitis occurrence, and 6-month survival via medical records review.

**Results:**

Among 1186 subjects with virologic failure, 35 (3.0%) were CrAg positive with median ART duration of 41 months (interquartile range, 10–84 months). Among 25 subjects with 6-month outcomes, 16 (64%) survived, 7 (28%) died, and 2 (8%) were lost. One survivor had suffered cryptococcal meningitis 2 years prior. Two others developed cryptococcal meningitis and survived. Five survivors were known to have received fluconazole. Thus, meningitis-free survival at 6 months was 61% (14/23). Overall, 91% (32/35) of CrAg-positive persons had viral load ≥5000 copies/mL compared with 64% (735/1151) of CrAg-negative persons (odds ratio, 6.0 [95% confidence interval, 1.8–19.8]; *P* = .001). CrAg prevalence was 4.2% (32/768) among those with viral loads ≥5000 copies/mL and 0.7% (3/419) among those with viral loads <5000 copies/mL.

**Conclusions:**

In addition to the CD4 threshold of <100 cells/μL, reflexive CrAg screening should be considered in persons failing ART in Uganda with viral loads ≥5000 copies/mL.

## Teaser

In addition to the CD4 threshold of <100 cells/μL, reflexive cryptococcal antigen screening should be considered in persons failing antiretroviral therapy with viral loads ≥5000 copies/mL.

Cryptococcal meningitis is responsible for 15% of AIDS-related deaths and is the leading cause of adult meningitis in sub-Saharan Africa [1, 2]. Annual global deaths from cryptococcosis are estimated at 181 100, with 75% of these deaths in sub-Saharan Africa [1]. Cryptococcal antigen (CrAg) is an independent predictor of mortality and can be detected in blood by latex agglutination a median of 3 weeks before developing initial symptoms of clinical cryptococcosis [3, 4]. CrAg screening is cost effective, has survival benefit, and is recommended by the World Health Organization (WHO) in persons with CD4 count <100 cells/μL, and can be considered at a CD4 count <200 cells/µL [5–9]. Among antiretroviral therapy (ART)–naive outpatient populations in low- and middle-income countries, the CrAg prevalence in 2014 averages 6% with some regional variations [1]. Among Ugandan ART-naive outpatients in 2004–2006 with CD4 count <100 cells/μL, the CrAg prevalence is 8.8% [10].

There is a substantial proportion of ART-experienced patients with virologic failure presenting with fulminant cryptococcosis [11]. During 2013–2017 in Uganda, nearly half of the patients presenting with cryptococcal meningitis were ART experienced [11]. These ART-experienced persons with virologic failure are at risk for cryptococcosis, yet they are not included within WHO CrAg screening guidelines’ high-risk category [5, 12]. As monitoring of those receiving ART is scaled up from CD4 to virologic monitoring in resource-limited settings, CD4-based algorithms for CrAg screening may not be readily applicable to ART-experienced populations. Baseline CD4 counts may be performed at HIV diagnosis but not routinely while monitoring HIV treatment. The 2018 Uganda HIV guidelines recommend CrAg screening in ART-naive persons, or those who fail treatment, with CD4 counts <100 cells/μL [13]. In the absence of CD4 monitoring, it is unknown whether to screen persons with virologic failure.

Given the substantial proportion of ART-experienced patients presenting with cryptococcosis, new viral load (VL)–based CrAg screening strategies may be useful. Uganda has also adopted the “test and treat” strategy, which will enroll more people on ART irrespective of their CD4 cell counts. In addition, we are now encouraging more virologic monitoring compared to CD4 monitoring. This creates a challenge for CrAg screening, which is based on CD4 cell counts. We conducted this study to determine the CrAg prevalence and clinical outcomes of CrAg-positive persons among ART-experienced people with virologic failure. We evaluated the potential threshold of virologic failure where CrAg screening should be considered, and the cost of CrAg screening at this threshold.

## METHODS

### Study Design, Setting, and Participants

This cross-sectional study was conducted at the Uganda National Health Laboratory Services (UNHLS), which performs centralized VL monitoring in Uganda (~95% of all HIV VL testing). The UNHLS processed a total of 843 020 VL tests for adults in 2017, of which 84 302 (10%) had unsuppressed viremia of ≥1000 copies/mL [14]. Dried blood spot and plasma samples from health facilities are delivered to the UNHLS via a hub transport system for HIV VL testing [15]. We retrospectively evaluated stored plasma samples of 1186 ART-experienced adults living with HIV (≥18 years) with suspected virologic failure with HIV VLs ≥1000 copies/mL. The sample size was estimated using the Buderer formula (using an absolute precision of 0.1, a 95% confidence interval [CI], and an estimated prevalence of 8.9% [from a CrAg study in Nigeria which had largely ART-experienced people] [16]) and assumed a sensitivity of 50%, giving us 1079 subjects to which we added a 10% margin for possible missing samples, making a total of 1186 subjects. We tested leftover plasma samples after VL testing from specimens collected during September 2017–January 2018. The samples from September 2017 to January 2018 were the most recent easily accessible and retrievable samples. We did not have stored samples of plasma for patients who had dried blood spots. CrAg lateral flow assay (LFA) testing on dried blood spots has lower sensitivity compared to plasma (unpublished data). CrAg testing was performed from March to July 2018 followed by retrospective records review to determine 6-month outcomes for the CrAg-positive patients.

### Study Procedures

Using the UNHLS database, we identified all patients with virologic failure and leftover plasma samples from the months of September 2017 to January 2018. From these we selected a random sample of 1186 patients with leftover samples using an auto-generated command in Stata version 14 software (StataCorp, College Station, Texas). The sampling technique used draws observations without replacement from a dataset. Quantitative RNA VLs on plasma had been done by UNHLS using the COBAS AmpliPrep/COBAS TaqMan system. For this study, we performed CrAg testing on stored plasma samples at UNHLS; CrAg testing was performed using the CrAg LFA (IMMY Inc, Norman, Oklahoma).

Subjects’ demographics and clinical data were obtained from the VL testing laboratory request forms. Outcome data were obtained by records review and/or contacting health facility clinicians. Additionally, clinicians were duly informed of the positive CrAg results. Patients who had not had any interaction with their respective clinics in the prior 6 months (from the point of their VL result) were defined as lost to follow-up. Meningitis-free survival was defined as having not suffered from clinical cryptococcal meningitis during the 6-month period after the HIV VL testing.

### Ethics Reviews

We obtained ethical review and approval from the Makerere University School of Medicine Research and Ethics Committee, the Uganda National Council of Science and Technology, and the University of Minnesota. A waiver for informed consent was obtained as CrAg testing was performed on leftover plasma samples, and the study posed no more than minimal risk to participants.

### Statistical Methods

Data analysis was primarily descriptive with variables summarized by mean with 95% CI, median with interquartile range (IQR), and number (percentage). We tested continuous variables via nonparametric Wilcoxon rank-sum test and categorical data via χ ^2^ test. We additionally used logistic regression to assess whether CrAg positivity was associated with quantitative HIV VL thresholds.

### Cost

Once CrAg prevalence was determined, we used the inverse to calculate the number needed to test to detect 1 CrAg-positive person. The cost of CrAg screening using the LFA in Uganda is $3.50 [17]. We multiplied the cost of screening by the number needed to test to identify the cost to detect 1 CrAg-positive person.

## RESULTS

### Prevalence of Cryptococcal Antigenemia in ART-experienced HIV-positive Patients With Virologic Failure

Overall, 368 174 adults living with HIV had VL testing from September 2017 to January 2018; 5348 patients had virologic failure and leftover plasma samples. We found 73 patients without leftover samples for CrAg testing. Of the randomly selected 1186 selected patients, 588 were from the Central region, 191 from the East, 147 from the North, and 260 from the West. These samples were from 96 of the 127 districts in Uganda. Table 1 shows a summary of demographic and clinical characteristics of the study population by CrAg result. Of the 1186 samples tested, 61% (724/1186) were collected from women. Participants had a mean age of 36 years (standard deviation, 10 years) and ranged from 18 to 75 years. Among 1186 ART-experienced persons with plasma VLs ≥1000 copies/mL, we identified 35 CrAg-positive persons equating to a CrAg prevalence of 3.0% (95% CI, 2.1%–4.1%). Among those with virologic failure, the median HIV-1 VL was 4-fold higher among CrAg-positive persons as compared with CrAg-negative persons (46 000 vs 12 000 copies/mL; *P* < .001).

**Table 1.** Clinical Characteristics of Participants by Plasma Cryptococcal Antigen Status

Characteristic	CrAg Positive (n = 35)	CrAg Negative (n = 1151)	*P* Value
Age, y, median (IQR)	35 (28–45)	36 (30–43)	.92
Women, no. (%)	20 (57)	704 (62)	.55
HIV-1 RNA, copies/mL, median (IQR)	46 400 (17 300–56 000)	12 000 (3020–69 800)	<.001
HIV-1 RNA ≥5000 copies/mL, no. (%)	32 (91)	735 (64)	.001
Duration of HIV therapy, No. (%)			
0.5–1 y	4 (12)	69 (6.7)	.22
>1–2 y	4 (12)	144 (14)	.77
>2–5 y	12 (36)	400 (39)	.80
>5 y	13 (39)	424 (41)	.86

*P* values are from Wilcoxon rank-sum test for medians and χ ^2^ test for proportions.

Abbreviations: CrAg, cryptococcal antigen; HIV-1, human immunodeficiency virus type 1; IQR, interquartile range.

### Distribution of HIV-1 Viral Load Among CrAg-positive Patients

**Figure 1.**
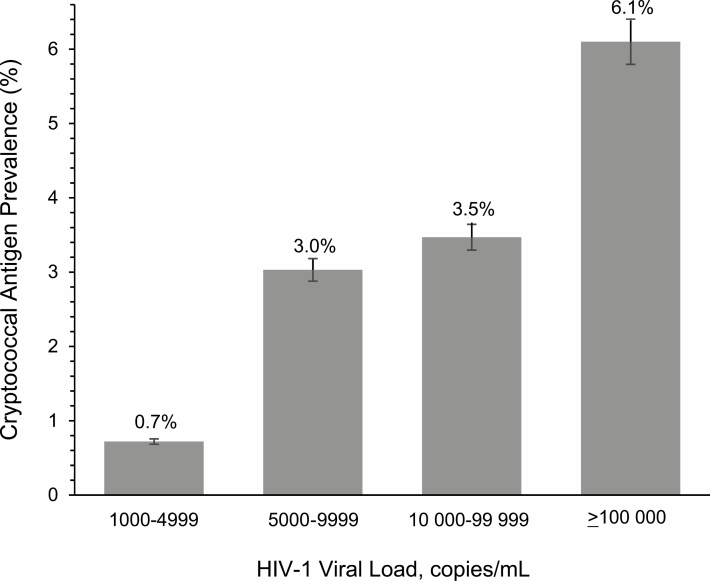
Cryptococcal antigen prevalence by human immunodeficiency virus type 1 (HIV-1) plasma viral load among Ugandans undergoing routine virologic monitoring on antiretroviral therapy.

Among the 1186 persons, 65% (767/1186) had VLs ≥5000 copies/mL. Overall, 91% (32/35) of CrAg-positive persons had VLs ≥5000 copies/mL compared with 64% (735/1151) of CrAg-negative persons (odds ratio, 6.0 [95% CI, 1.8–19.8]; *P* = .001). CrAg prevalence increased among higher VLs, with 4.2% (32/768) CrAg positivity among those with ≥5000 copies/mL vs 0.7% (3/419) CrAg positivity among those with <5000 copies/mL. Figure 1 displays prevalence of CrAg positivity with respect to HIV VL.

### Outcomes of CrAg-positive Patients With Virologic Failure

Among the 35 CrAg-positive persons, we obtained 6-month survival data for 25 persons. We did not get outcome data on the other 10 CrAg-positive persons because efforts to contact their respective clinicians or to access their medical files were futile. Of the 25 CrAg-positive persons with outcome data, 16 (64%) were known to be alive, 7 (28%) were confirmed dead, and 2 (8%) were lost to follow-up.

Of the 25 CrAg-positive persons, only 1 person had a history of treatment for cryptococcal meningitis 2 years prior and was taking fluconazole for secondary prophylaxis. Of the 25 CrAg-positive persons, 17 were getting their first ever CrAg test. Five survivors were known to have been CrAg tested and received preemptive therapy with fluconazole around the time of their VL result. Another 2 patients who developed cryptococcal meningitis during these 6 months were treated and survived. Thus, meningitis-free survival at 6 months was 14 of 23 (61% [95% CI, 35%–76%]). Absent fluconazole receipt (n = 19), 6-month meningitis-free survival was 8 of 19 (42% [95% CI, 20%–67%]).

We also did an analysis including those who were lost to follow-up. We used 6-month outcome findings from a CrAg-positive cohort in Uganda that found a 14% mortality rate, 77% meningitis-free survival, and 9% progression to meningitis [10]. If we applied this to the 10 without survival data, we would have 8 who survived meningitis free, 1 who died, and 1 who developed cryptococcal meningitis. This would then make a 63% (22/35) meningitis-free survival rate. If we were to consider that all the 10 without survival data died, then the meningitis-free survival rate would be 40% (14/35).

We determined the likely outcome of those lost to follow-up (including those without outcome data) by comparing their sex, median ages, and VLs with those who survived and died. We used the Fisher exact test for the sex comparison across the 3 groups and found no significant difference (*P* = .42). The Kruskal-Wallis test was used to compare the medians for age and VL across the 3 groups and the test was not significant for both age (*P* = .42) and VL (*P* = .10). Therefore, the likely outcome of the individuals lost to follow-up may be similar to those who were accounted.

### Cost of CrAg Screening

We identified 4.2% CrAg prevalence among persons with a HIV VL ≥5000 copies/mL. Thus, the number needed to test to detect 1 CrAg-positive person is 25. At a cost of $3.50 for the CrAg LFA [17], the cost to detect 1 CrAg-positive person among those with virologic failure is $87.50.

## Discussion

The CrAg prevalence was 3.0% in ART-experienced HIV patients with virologic failure and 4.2% among those with ≥5000 HIV RNA copies/mL. To our knowledge, this is the first study to evaluate CrAg prevalence in ART-experienced patients with virologic failure, irrespective of CD4 count. This CrAg prevalence rate was similar to that in ART-experienced populations in studies in South Africa (2.8%), Brazil (3.1%) [18, 19], and in Ethiopia (4.1%) [20] but lower than that in studies in Nigeria (8.9%) and Ethiopia (8.4%), though all these studies included participants responding to ART or did not report VLs [12, 16]. CrAg positivity was shown to significantly increase with higher VLs, indicating a higher risk for cryptococcosis in people with more fulminant virologic failure. We found an all-cause 6-month meningitis-free survival rate of 61% among CrAg-positive persons, of whom at least 6 survivors received fluconazole therapy. Absent this fluconazole therapy, meningitis-free survival was approximately 40%, although the 95% CI was wide.

We calculated that it would cost $87.50 to detect 1 CrAg-positive person if screening is performed among persons with VLs of ≥5000 copies/mL. In 2018, Uganda had 951 690 adult HIV VL samples tested; 858 426 were suppressed, 6661 tests were rejected, and 86 603 were virologic failures (VL ≥1000 copies/mL) [14]. From our sample, one could assume that 64% of those with virologic failure had a VL ≥5000 copies/mL. Thus 55 426 persons would be screened per year, and 2327 would be CrAg positive. Thus, of the 951 690 total VL samples received, the additional cost would be approximately $194 000 in total, or $0.20 per VL sample received.

As HIV programs ramp up “test and treat” and scale up virologic monitoring, we have observed increasing proportions of persons with cryptococcal meningitis presenting as ART-experienced persons with undetected virologic failure [11, 21]. WHO guidelines recommend CrAg screening for persons with CD4 count <100 cells/μL prior to initiating ART [9, 22]; however, no guidelines exist for ART-experienced persons. Given that virologic monitoring is emphasized more than CD4 monitoring in this population, using a VL threshold to identify who needs CrAg screening becomes more pertinent. The guidelines acknowledge that CrAg screening in ART-experienced persons needs to be further evaluated. Based on our findings, in the absence of CD4 monitoring, we would recommend CrAg screening among persons with virologic failure ≥5000 copies/mL as this threshold where we found a CrAg prevalence of 4.2%.

The study had limited access to patient medical information, thus missed some clinically relevant data, such as CD4 cell count, which could have been useful in further characterizing the population of interest. This could also have introduced information bias in characterizing patients as meningitis free. The potential seasonal distribution of cryptococcal infections may have influenced prevalence results. While we only tested plasma specimens, whether dried blood spots would have a different CrAg prevalence is unclear as the CrAg LFA sensitivity is lower in dried blood spots than in plasma due to the extraction process (unpublished data). Viral load testing is being scaled up replacing CD4 monitoring, yet absolute CD4 cell counts provide the risk stratification to target evaluation for opportunistic infections, including CrAg screening. The cost of CD4 testing (~$6) is more than the cost of CrAg testing (≤$4), so from a program implementation perspective, using CD4 testing to then narrow the pool to select for CrAg testing is not efficient, unless a CD4 is already being performed [17, 23]. We should consider expanding the threshold for CrAg testing by including those with VL ≥5000 copies/mL, which would decrease the amount of CrAg testing by 35% (419/1186) yet still detect >90% (32/35) of CrAg-positive persons. The limitations of this approach are that it would be dependent on the turnaround time for VL. Another limitation of the study was that we did not perform CrAg titers. This information could be useful to determine if persons with low plasma CrAg LFA titers ≤1:20 [24] may respond well to prompt switching to second-line ART only. The strengths of the study include generalizability of the results because we had access to samples from all regions of the country. This study also provided an opportunity to pilot implementation of the Uganda CrAg screening guidelines, which now include CrAg screening of individuals with virologic failure and those with a positive symptom screen in the advanced HIV disease pathway [13]. Further studies in different geographical areas where cryptococcosis is common are warranted to characterize the epidemiology of cryptococcal disease among persons failing ART and to evaluate the cost effectiveness of using VL testing as an entry point to CrAg screening in this population. Prospective studies on the implementation of such a screening strategy and clinical outcomes with fluconazole preemptive therapy are needed to further characterize this potential strategy to reduce AIDS-related deaths among persons with virologic failure.

This study shows that ART-experienced people failing HIV therapy are at risk for disseminated cryptococcal antigenemia and eventually cryptococcal meningitis or death and demonstrates the feasibility of linking CrAg screening to VL monitoring. To further avert cryptococcosis among individuals with virologic failure, WHO should recommend CrAg screening in persons with virologic failure with VLs ≥5000/mL. Cost-effective studies are needed to establish whether CrAg screening based on VL thresholds (alongside CD4 counts) would be feasible. Studies to determine the association of virologic failure independent of CD4 and CrAg are needed. Prospective studies among ART-experienced populations to further evaluate the optimal VL threshold for CrAg testing, timing of ART switch, implementation of this strategy, and the potential benefit of CrAg screening are warranted.

## Notes

***Disclaimer.*** The views expressed in this publication are those of the authors and not necessarily those of the African Academy of Sciences (AAS), the New Partnership for Africa’s Development Planning and Coordinating Agency (NEPAD), the Wellcome Trust, or the government of the United Kingdom.

***Financial support.*** This research was supported by the National Institutes of Health Fogarty International Center (grant numbers D43TW009345 to E. M. and K01TW010268 to J. R.) and the National Institute of Allergy and Infectious Diseases (grant numbers K23AI138851 to R. R. and U01AI125003 to D. B. M., D. R. B., R. R.). D. B. M. is supported by the Developing Excellence in Leadership, Training and Science (DELTAS) Africa Initiative (grant number DEL-15-011 to Training Health Researchers Into Vocational Excellence in East Africa [THRiVE-2]). The DELTAS Africa Initiative is an independent funding scheme of the AAS’s Alliance for Accelerating Excellence in Science in Africa and supported by NEPAD with funding from the Wellcome Trust (grant number 107742/Z/15/Z) and the UK government.

***Potential conflicts of interest.*** The authors: No reported conflicts of interest. All authors have submitted the ICMJE Form for Disclosure of Potential Conflicts of Interest.
